# Frequency of respiratory pathogens and SARS‐CoV‐2 in canine and feline samples submitted for respiratory testing in early 2020

**DOI:** 10.1111/jsap.13300

**Published:** 2021-01-31

**Authors:** H. T. Michael, T. Waterhouse, M. Estrada, M. A. Seguin

**Affiliations:** ^1^ IDEXX Laboratories, Inc 1 IDEXX Drive Westbrook ME 04092 USA; ^2^ IDEXX Laboratories, Inc 2825 KOVR Dr West Sacramento CA 95605 USA

## Abstract

**Objectives:**

The emergence of the 2019 novel coronavirus (SARS‐CoV‐2) has necessitated evaluation of the potential for SARS‐CoV‐2 infection in dogs and cats. Using a large data set, we evaluated the frequency of SARS‐CoV‐2 and other respiratory pathogens in samples submitted for respiratory testing from mid‐February to mid‐April 2020.

**Materials and Methods:**

A SARS‐CoV‐2 real‐time PCR was developed and validated. A subset of canine and feline samples submitted for respiratory pathogen panel testing to reference laboratories in Asia, Europe, and North America were also tested for SARS‐CoV‐2. The frequency of respiratory pathogens was compared for the February–April period of 2020 and 2019.

**Results:**

Samples from 4616 patients were included in the study and 44% of canine and 69% of feline samples were PCR positive with *Mycoplasma cynos* and *Bordetella bronchiseptica* and *Mycoplasma felis* and feline calicivirus, respectively. No SARS‐CoV‐2 infections were identified. Positive results for respiratory samples were similar between years.

**Clinical Significance:**

The data in this study suggest that during the emergence of the SARS‐CoV‐2 pandemic in early 2020, respiratory diseases in tested pet cats and dogs were caused by common veterinary pathogens and that SARS‐CoV‐2 infections in dogs and cats are rare.

## INTRODUCTION

With the emergence of the 2019 novel coronavirus (SARS‐CoV‐2) and the resulting pandemic of human coronavirus disease 2019 (COVID‐19) there has been public concern about the potential for SARS‐CoV‐2 infections in other species. The SARS‐CoV‐2 virus is a betacoronavirus and is most similar to a bat coronavirus (Wu *et al*. 2020) but also shares many similarities to some pangolin betacoronaviruses (Zhang *et al*. 2020). Research suggests that experimental infection can occur in cats, golden hamsters, non‐human primates, and ferrets (Chan *et al*. 2020, Kim *et al*. 2020, Munster *et al*. 2020, Sia *et al*. 2020, Shi *et al*. 2020a). SARS‐CoV‐2 infections have been found in mink at mink farms in the USA and the Netherlands (Oreshkova *et al*. 2020, United States Department of Agriculture 2020a). Potential transmission from infected mink to workers on farms has also been reported (Oreshkova *et al*. 2020, Oude Munnink *et al*. 2020). The potential for infection in pet dogs and cats and spread between pets or between pets and their owners have been areas of concern. By June 2020, 7 million cases of Covid‐19 were diagnosed worldwide, but fewer than 20 confirmed cases of SARS‐CoV‐2 infections had been reported in domestic dogs and cats worldwide by that time (American Veterinary Medical Association 2020). Transmission of SARS‐CoV‐2 to pet dogs and cats is believed to be rare and transmission is believed to be principally from infected humans to their pets (American Veterinary Medical Association 2020). Most of the infections identified in cats have been associated with clinical signs but some dog infections have not (American Veterinary Medical Association 2020). These veterinary SARS‐CoV‐2 infections have garnered attention in the popular press, leading to fear and confusion for pet owners, animal shelters and veterinarians about respiratory illness in dogs and cats, risk of infections in household pets and the potential for zoonotic transmission.

A wide variety of pathogens are known to cause infectious respiratory disease in dogs and cats (Cohn 2011, Priestnall *et al*. 2014). As it is not possible to differentiate between pathogens based solely on clinical signs, further testing is required to identify the pathogen responsible and guide specific therapy. Additionally, some microorganisms, such as *Mycoplasma* species, can be present as commensals or as secondary pathogens, requiring correlation of both testing and clinical signs (Haesebrouck *et al*. 1991, Lee‐Fowler 2014). Adding to public confusion about coronaviruses, dogs can be infected with the canine respiratory coronavirus (CrCoV), which is a different betacoronavirus (Erles & Brownlie 2008). To assess the frequency of SARS‐CoV‐2 infections and to identify patterns of canine and feline respiratory pathogens in patients undergoing testing for respiratory pathogens, we designed a study using the large data sets and samples available to IDEXX Laboratories to develop a snapshot of respiratory pathogens, including SARS‐CoV‐2, in samples submitted for respiratory disease testing during the early months of the COVID‐19 pandemic and the same period for the year prior.

## METHODS

### Patient inclusion

For the surveillance of SARS‐CoV‐2 and other respiratory pathogens in early 2020, a convenience sample of canine and feline respiratory samples submitted to IDEXX Reference Laboratories for commercially available respiratory real‐time PCR panels in Asia (mid‐February to mid‐March 2020), Europe (mid‐March to mid‐April 2020) and North America (mid‐February to mid‐April 2020) was enrolled in the study. Conjunctival and deep pharyngeal swabs were submitted for each patient. If multiple samples were submitted for an individual patient during the study window, only the first sample was included in the analysis. Results were anonymised before enrolment in the study and no additional clinical information was gathered.

For evaluation of year‐over‐year differences in pathogen frequency, 5000 canine and 5000 feline respiratory PCR panel results were selected for each 2019 and 2020. Real‐time PCR tests (Feline Upper Respiratory RealPCR™ Panel or Canine Respiratory RealPCR Panel, IDEXX Laboratories, Inc.) were performed at IDEXX Reference Laboratories. Conjunctival and deep pharyngeal swabs were submitted for each patient. As above, results from duplicate samples from the same patient were removed. Samples chosen for the year over year comparison were selected by simple random sampling to reflect the population submitted for respiratory testing worldwide. Random selection was performed using the sample n function in R version 3.6.2 (R Core Team 2019) from the results available from the period of February, March, and April for 2019 and 2020 from the same geographic regions included in the early 2020 surveillance (above). Samples from the 2020 surveillance population may be included within the year‐over‐year comparison. Only PCR panel results were collected for analysis. No identifying information or demographic data was obtained.

### PCR methodology

The basic methodology for all real‐time PCR tests (respiratory panels and SARS‐CoV‐2) is the same and has been previously described (McManus *et al*. 2014, Schulz *et al*. 2014, Litster *et al*. 2015). All primers, probes and sequencing primers for the respiratory pathogens and SARS‐CoV‐2 are proprietary. Conjunctival and deep pharyngeal swabs from each sample were pooled for nucleic acid extraction. RNA and DNA were extracted using a guanidinium thiocyanate‐based reagent (Boom *et al*. 1990) and isolated using magnetic bead‐based nucleic acid extraction on the KingFisher Flex 96 (Thermofisher) automated platform. For RNA samples, cDNA synthesis was performed using random primers and oligoDT. PCR reactions were run on a standardised high‐throughput platform using the LightCycler 480 (Roche Molecular Systems). Cycle thresholds of 39.9 or lower were considered positive and 40 or greater was considered negative for all real‐time PCR tests. Controls were used to determine if the results of the run should be accepted or discarded due to nucleic acid contamination, insufficient sample or sample degradation, or failure of the reaction due to reagent, technical or pipetting failures. All real‐time PCR were run with seven quality controls including: (1) PCR‐positive controls to assess functionality of the PCR run, (2) PCR‐negative controls (blank) to confirm absence of nucleic acid contamination in the reagents, (3) negative extraction controls (phosphate‐buffered saline blank) to confirm the absence of nucleic acid contamination during the extraction process, (4) DNA pre‐analytical quality control targeting the host 18S rRNA gene complex to assess DNA quality and integrity for the patient sample following extraction, (5) RNA pre‐analytical quality control targeting the host 18s rRNA gene complex to ensure RNA quality and integrity following extraction and successful reverse transcription, (6) an internal positive control of synthetic nucleic acids spiked into the lysis solution to verify successful a PCR run and absence of PCR inhibitory substances as a carryover from the sample matrix, and (7) an environmental contamination monitoring control to ensure no environmental nucleic acid contamination in the laboratory. Microorganisms and target genes included in the canine and feline respiratory RealPCR panels are in Tables S1 and S2.

### SARS‐CoV‐2 real‐time PCR development and validation

A SARS‐CoV‐2 real‐time PCR assay (now commercially available as the IDEXX SARS‐CoV‐2 RealPCR®, IDEXX Laboratories) was developed with proprietary primers targeting the SARS‐CoV‐2 nucleocapsid phosphoprotein gene (NC_045512). As a further quality assurance, three PCR assays targeting the SARS‐CoV‐2 nucleocapsid protein and developed by the Center for Disease Control (CDC assays) (Center for Disease Control and Prevention 2020a, 2020b) were run in parallel for all samples. General PCR protocol was as above for real‐time PCRs performed at IDEXX Laboratories. Primer and assay development was based on the CDC protocols (Center for Disease Control and Prevention 2020a, 2020b) and adapted for use on the LightCycler 480 used for all IDEXX RealPCR tests. Thermal cycling conditions for the SARS‐CoV‐2 real‐time PCR included 1 activation cycle (2 minutes at 50°C then 10 minutes at 95°C), 45 amplification cycles (10 seconds at 95°C, 30 seconds at 60°C and 1 seconds at 72°C) and 1 cooling cycle (10 seconds at 40°C). Validation was performed to a previously published industry‐standard methodology for probe‐based real‐time PCR (Livak *et al*. 1995) that all IDEXX PCR tests must pass to ensure uniform performance characteristics necessary to run PCR tests in parallel for individual samples. The validation criteria included amplification efficiency, dynamic range, analytical sensitivity (at least 10 molecules per PCR reaction, within run and in between run reproducibility (CP values: <3% and with linearized values: <20%), r^2^ value of the curve (0.993 or better), signal to noise ratio of the fluorophore release in positive PCR reactions (10‐fold) and confirmation of analytical specificity by resequencing positive clinical material using proprietary external sequencing primers to verify that there was no drift within the assay or primers.

Performance of the SARS‐CoV‐2 test was tested on 48 clinically characterised human isolates (32 SARS‐CoV‐2 positive samples and 16 negatives). The human samples were tested with the SARS‐CoV‐2 real‐time PCR test and the three CDC tests (as described above) and compared with the original clinical results from HUMAN Diagnostics Worldwide (Wiesbaden, Germany).

Cross‐reactivity of the SARS‐CoV‐2 PCR test was evaluated with canine respiratory coronavirus (CrCoV), canine enteric coronavirus (CeCoV), feline enteric coronavirus (FeCoV) and equine coronavirus (ECoV). Patient samples determined to be positive for one of these veterinary coronaviruses using the appropriate, commercially‐available RealPCR test (IDEXX Laboratories) were also tested with the SARS‐CoV‐2 real‐time PCR. The human patient samples (described above) were also tested with the same veterinary coronavirus RealPCR tests to determine if there was cross‐specificity of the veterinary tests with SARS‐CoV‐2.

Feline and canine patient samples submitted for respiratory pathogen testing and included in the study (described above) had SARS‐CoV2 testing run on remaining nucleic acids following the respiratory pathogen testing. The SARS‐CoV‐2 real‐time PCR (described above) and the three CDC assays (CDC 2020) were run in parallel on all samples. Results for the SARS‐CoV‐2 testing during this study were for research use only and were not reported to the submitting veterinarian.

### Statistics

The same analyses were used on the surveillance samples from early 2020 and the year‐over‐year comparison samples. The analysis was run in R version 3.6.2. Each test in the real‐time PCR panels was then assigned to a specific pathogen group to account for slightly different names for individual pathogen tests reported in different geographic regions. Once the assays were grouped, a contingency table for pathogen groups and test results was constructed. Patients that tested positive for more than one pathogen were flagged for additional analysis.

The positivity rate for a pathogen group was calculated as the proportion of patients tested that received positive results. The rate of patients who tested positive for more than one pathogen group was calculated as the proportion of patients tested that were flagged as testing positive for more than one pathogen. Exact binomial 95% confidence intervals (CI) were calculated for all positivity estimates. Change in the positivity rate was determined by subtracting the overall 2020 rate from the overall 2019 rate. Statistical significance of year over year rate changes was performed using the Holm‐Bonferroni method.

## RESULTS

### Performance of the SARS‐CoV‐2 real‐time PCR test

Given the paucity of veterinary SARS‐CoV‐2 infections, the performance of our real‐time PCR SARS‐CoV‐2 test was assessed and validated with 48 clinically characterised isolates from human patients. The SARS‐CoV‐2 real‐time PCR accurately identified all human samples (32 SARS‐CoV‐2 positives and 16 SARS‐CoV‐2 negatives) for 100% specificity and 100% sensitivity on these specimens. The SARS‐CoV‐2 assay met or exceeded all pre‐identified specifications for reproducibility and reliability. Sensitivity and specificity were determined to >95% with linearity >5 log values, an *r* ^2^ >0.993 and a signal to noise ratio >10 fluorescent units.

Cross‐specificity testing to rule out false positives caused by other veterinary coronaviruses was performed using veterinary patient samples that had tested positive at IDEXX Reference Laboratories using commercially available PCR tests for the CrCoV (30 samples), CeCoV (30 samples), FeCoV (30 samples) and ECoV (two samples). None of these samples had a positive result with the SARS‐CoV‐2 real‐time PCR. None of the 55 human patient isolates (36 SARS‐CoV‐2 positive and 19 SARS‐CoV‐2 negative) tested were positive for the CrCoV, CeCoV, FeCoV or ECoV.

### Monitoring of respiratory pathogens and SARS‐CoV‐2 during February to April 2020

**FIG 1:**
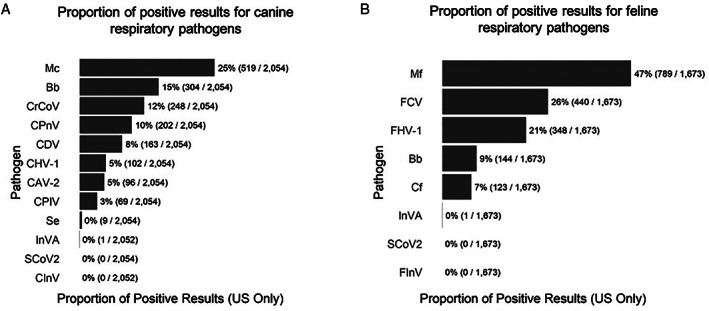
Proportion of positive PCR results for respiratory pathogens in (A) canine and (B) feline samples from the US in mid‐February to mid‐March 2020. Mc *Mycoplasma cynos*; Bb *Bordetella bronchiseptica*; CrCoV canine respiratory coronavirus; CPnV canine pneumovirus; CDV canine distemper virus; CHV‐1 canine herpesvirus type 1; CAV‐2 canine adenovirus type 2; CPIV canine parainfluenza virus; Se *Staphylococcus equi*; InVA influenza A virus (H1N1, H3N2, H3N8 and H7N2); SCoV2 SARS coronavirus 2; CInV H3N2 canine influenza virus; Mf *Mycoplasma felis*; FCV feline corona virus; FHV‐1 feline herpes virus type 1; Cf *Chlamydophila felis*; FInV H7N2 influenza virus

A total of 4616 patients (2150 dogs and 2466 cats) were included in the surveillance SARS‐CoV‐2 on samples submitted for respiratory disease panels. The geographic distribution of samples is shown in Table 1. For dogs, positive results for at least one respiratory pathogen were identified in 44.1% (949/2150) of patient samples (Table 2). No SARS‐CoV‐2 positive results were identified in any of the canine samples. There were insufficient numbers of test results for the interpretation of individual pathogen frequencies for samples outside of the USA, but those data are presented in Tables S3 and S4. In the USA samples, *Mycoplasma cynos*, was the most common microorganism and was identified in over 25% of the samples Fig 1A). Infection with more than one pathogen occurred in 16.9% (365/2150) of canine patients and the most common co‐infection was with *M. cynos* and *Bordetella bronchiseptica* (129 cases). For cats, at least one pathogen was identified in 69.5% (1713/2466) of samples (Table 2). No feline samples were positive for SARS‐CoV‐2 in this population.

**Table 1:** Geographic origin of samples included in the SARS‐CoV‐2 surveillance population in early 2020

Geographic region	Canine	Feline
Asia		
South Korea	35	171
Singapore	0	3
Europe		
Germany	40	465
Austria	2	24
Italy	2	24
Denmark	6	21
Norway	4	20
Sweden	0	20
Finland	0	20
Netherlands	2	18
North America		
USA	2054	1673
Canada	4	5
Mexico	1	2
Total	2150	2466

**Table 2:** Proportion of samples in the SARS‐CoV‐2 surveillance population in early 2020 with at least one positive result on the respiratory PCR panel

Geographic region	Canine positive results		Feline positive results	
Asia	28.6%	10/35	60.3%	105/174
Europe	25.0%	14/56	68.5%	419/612
N America	44.9%	925/2059	70.8%	1189/1680
Total	44.1%	949/2150	69.5%	1713/2466

*Mycoplasma felis* and feline calicivirus (FCV) were the most common organisms identified in the USA samples (Fig 1B). Infection with more than one pathogen was identified in 26.3% (652/2467), most commonly co‐infection with *M. felis* and FCV (354 cases).

### Year‐over‐year comparison of respiratory pathogen frequency

**FIG 2:**
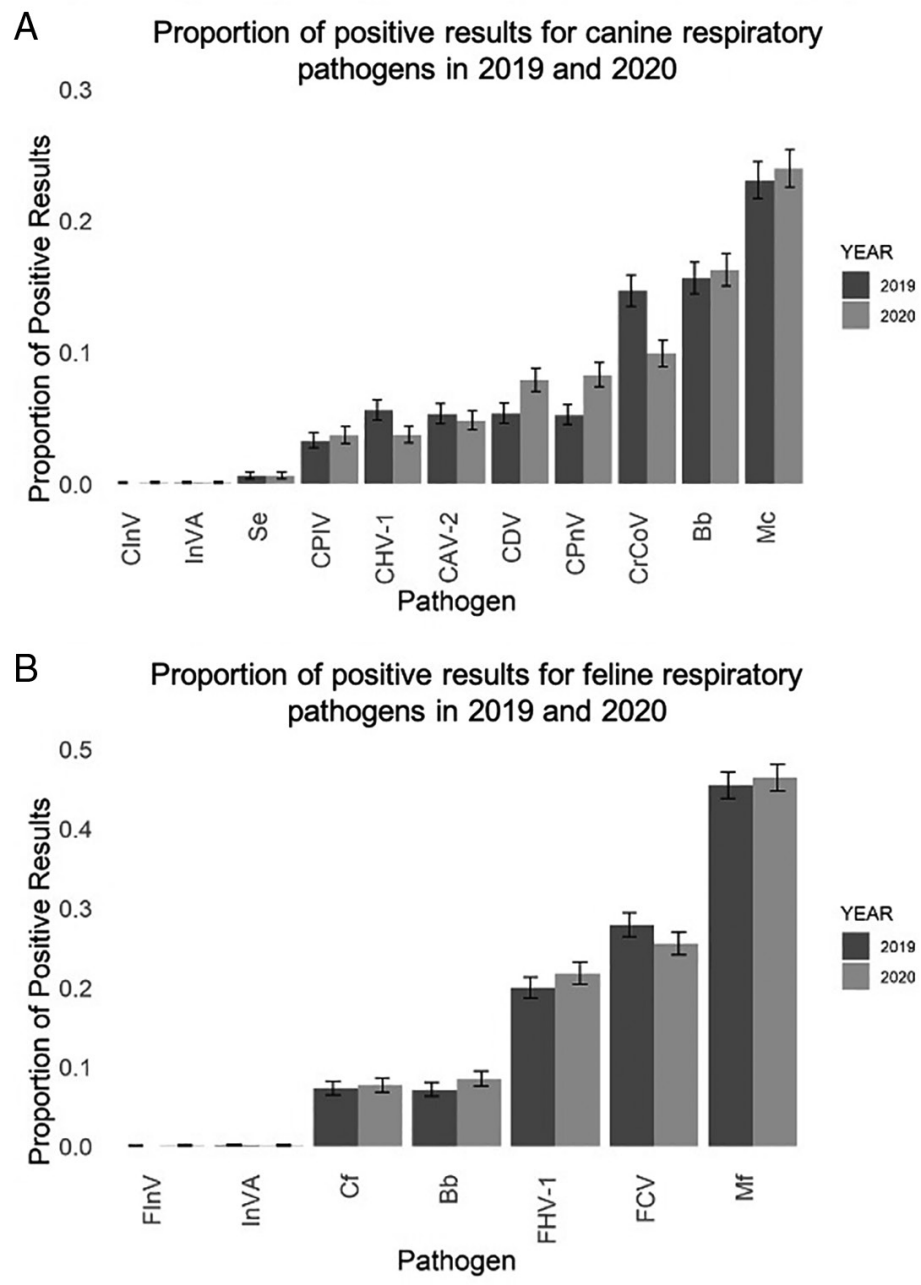
Comparison of the proportion of individual respiratory pathogens from February through April in 2019 and 2020. (A) Proportion of positive results from 5000 canine respiratory panels submitted in each year. (B) Proportion of positive results from 5000 feline upper respiratory panels submitted each year. Error bars show 95% confidence intervals. Mc *Mycoplasma cynos*; Bb *Bordetella bronchiseptica*; CrCoV canine respiratory coronavirus; CPnV canine pneumovirus; CDV canine distemper virus; CHV‐1 canine herpesvirus type 1; CAV‐2 canine adenovirus type 2; CPIV canine parainfluenza virus; Se *Staphylococcus equi*; InVA influenza A virus (H1N1, H3N2, H3N8 and H7N2); SCoV2 SARS coronavirus 2; CInV H3N2 canine influenza virus; Mf *Mycoplasma felis*; FCV feline corona virus; FHV‐1 feline herpes virus type 1; Cf *Chlamydophila felis*; FInV H7N2 influenza virus

Since the frequency and type of respiratory infections can vary both from year to year and season to season, we compared the data from early 2020 with the same timepoint in 2019 to provide context if the early months of the Covid‐19 pandemic were causing large shifts in respiratory disease pathogens or the frequency of positive results in the tested population. The proportion of total positive tests was slightly higher in 2020 for both dogs and cats than in 2019 for the USA and Europe and decreased for Asia (Tables 3 and 4). The differences in total positive rate were significantly different only for Europe. There is variation in the number of tests included for regions for the 2 years due to differences in the proportion of respiratory PCR tests submitted from these regions during the study periods each year. Only the USA had sufficient data for statistical characterizations of individual pathogens. Within the USA, the frequency of individual pathogens for dogs and cats were similar from year to year (Fig 2). Notable exceptions were year‐over‐year increases in canine pneumovirus (CPnV) from 4.9% (4.3–5.5 95% CI) in 2019 to 9.4% (8.7–10.3 95% CI) in 2020 and in canine distemper virus from 5.0% (4.4–5.6 95% CI) in 2019 to 7.6% (6.86–8.32 95% CI) in 2020 and a decrease in the frequency of canine respiratory coronavirus (CrCoV) from 13.9% (12.9–14.8 95% CI) in 2019 to 9.5% (8.7–10.3 95% CI) in 2020 (Fig 2A). Feline respiratory pathogen frequency was similar for both years (Fig 2B).

**Table 3:** Year‐over‐year change in the proportion of canine positive respiratory PCR tests for at least one pathogen in February through April in 2019 and 2020

Geographic region	2019		2020		YoY change	Padj
Asia	52.0%	40/77	36.4%	20/55	−15.6%	0.3
Europe	5.2%	5/96	20.6%	34/165	+15.4%	.004
N America	47.5%	2293/4827	48.2%	2308/4780	+0.7%	1

YoY Year‐over‐year; padj P adjusted value.

**Table 4:** Year‐over‐year change in proportion of feline positive respiratory PCR results for at least one respiratory pathogen in February through April 2019 and 2020

Geographic region	2019		2020		YoY change	Padj
Asia	56.9%	149/262	59.6%	129/218	+2.8%	1
Europe	57.9%	733/1265	64.9%	984/1517	+6.9%	.001
N America	69.6%	2417/3473	70.0%	2285/3265	+0.4%	1

YoY Year‐over‐year; padj P adjusted value.

## DISCUSSION

These data indicate that positive respiratory PCR tests were caused by the typical veterinary pathogens during the early months of the COVID‐19 pandemic in early 2020. The failure to identify any SARS‐CoV‐2 infections in over 4500 respiratory samples during the surveillance study period supports the conclusion that SARS‐CoV‐2 infections in pet dogs and cats are rare. During this time, many of the regions included in the study had local restrictions on movement and veterinary practice which impacted both the type and quantity of veterinary care sought by owners in early 2020. Although the year‐over‐year comparisons suggest that the frequency of positive tests and of most pathogens were similar, we cannot entirely exclude that these restrictions may have introduced some selection bias into the early 2020 populations. Therefore, the early 2020 data should not be construed as a true prevalence of respiratory pathogens in the tested population during that period.

Year‐over‐year changes in the frequency of individual pathogens suggest an increase in canine pneumovirus and decrease in canine respiratory coronavirus, both of which are relatively new respiratory pathogens (Erles & Brownlie 2008, Renshaw *et al*. 2010, Mitchell *et al*. 2013, Priestnall *et al*. 2014, Day *et al*. 2020). Additional study to determine if this represents a persistent or temporary change in the CrCoV prevalence would be needed to draw any conclusions about the cause of the decrease. However, the similarities between the data from 2020 and 2019 suggest that there have not been large shifts in feline and canine respiratory pathogens during our study period.

To better understand the frequency of SARS‐CoV‐2 infection in pet dogs and cats and other veterinary species, further surveillance is needed. As the number of human Covid‐19 cases has risen worldwide, there have been increasing numbers of isolated reports of confirmed SARS‐CoV‐2 infections in animals. Following the end of the study period in early 2020, the number of cases of Covid‐19 in the USA increased dramatically. Although no SARS‐CoV‐2 infections were identified in our data set during the study period, cases of naturally occurring infections in pet dogs (two) and cats (five) have now been identified in the United States with the SARS‐CoV‐2 real‐time PCR test described hereafter it became commercially available (IDEXX Laboratories 2020, United States Department of Agriculture 2020b). Cases reported in the USA are confirmed with additional PCR and sequencing by the United States Department of Agriculture (USDA) National Veterinary Laboratories (Newman *et al*. 2020) according to their protocols. By the late September 2020, the USDA reported that 39 cases of SARS‐CoV‐2 in pet dogs and cats (18 dogs and 21 cats) had been confirmed in the USA as well as cases in zoo animals and farmed mink (United States Department of Agriculture 2020b). Full clinical information is not available for all cases. Clinical presentations for the animals have varied and not all have had clinical signs. Of the five cats tested with the SARS‐CoV‐2 RealPCR test, four had respiratory signs (mild to severe) and one had oral ulcerations without respiratory signs (IDEXX Laboratories 2020). Although it has been presumed that SARS‐CoV‐2 will cause respiratory disease in dogs and cats, there is increasing evidence of extra‐respiratory disease in human patients, including gastrointestinal effects (Gu *et al*. 2020, Jin *et al*. 2020), acute kidney injury (Pan *et al*. 2020, Su *et al*. 2020), cardiac injury (Shi *et al*. 2020b) and thrombosis (Bikdeli *et al*. 2020, Klok *et al*. 2020). As the number of SARS‐CoV‐2 infections confirmed in dogs and cats accumulate over time, further characterisation of clinical manifestations will be needed both for clinical diagnosis and care and for monitoring infection in the dog and cat population.

These data from early 2020 support and complement the hypothesis that naturally occurring SARS‐CoV‐2 infections are uncommon in dogs and cats. To date, cases of SARS‐CoV‐2 in pets are believed to represent reverse zoonosis (American Veterinary Medical Association 2020). There is no evidence at this time of transmission from pets to humans. To better understand the potential for transmission of SARS‐CoV‐2 within households, and the potential for subclinical cases in dogs and cats, further study will be needed as additional SARS‐CoV‐2 infections are identified in dogs and cats. The current COVID‐19 pandemic does not remove the necessity to test for common veterinary respiratory pathogens in animals with appropriate clinical signs. The respiratory pathogens identified by PCR in early 2020 were similar to those from 2019, despite the COVID‐19 pandemic. These data suggest there is currently no need for widespread SARS‐CoV‐2 testing in the dog and cat population since naturally occurring clinical infections are rare in dogs and cats. Practitioners should continue to consider and test for common respiratory pathogens before SARS‐CoV‐2 infection is considered in pet dogs and cats with respiratory signs.

### Conflict of interest

All authors are employees of IDEXX Laboratories.

## Supporting information


**Table S1.** Bacteria and virus gene targets included in the canine respiratory disease panels.

**Table S2.** Bacteria and virus gene targets included in the feline upper respiratory panel.

**Table S3.** Counts of individual respiratory microorganisms identified by PCR for feline patients outside of the USA included in the SARS‐CoV‐2 surveillance study in early 2020.

**Table S4.** Counts of individual respiratory microorganisms identified by PCR for canine patients outside of the USA included in the SARS‐CoV‐2 surveillance study in early 2020.

**Table S5.** Geographic origin of samples included in the year‐over‐year comparison.
