# Supplementary material for: Frequency of respiratory pathogens and SARS‐CoV‐2 in canine and feline samples submitted for respiratory testing in early 2020
Source: J Small Anim Pract. 2021 Jan 31;62(5):336–42. doi: 10.1111/jsap.13300 (PMC8014115; doi:10.1111/jsap.13300)
Supplement: Supplementary file 1 — Table S1. Bacteria and virus gene targets included in the canine respiratory disease panels. [file JSAP-62-336-s002.docx]

| **Pathogens tested for in the canine respiratory disease panel** | **Gene Target** |
| --- | --- |
| *Bordetella bronchiseptica*  Canine adenovirus type 2  Canine distemper virus  Canine herpesvirus type 1  Canine pneumovirus  Canine parainfluenza virus  H3N2 canine influenza virus  Influenza A virus (H1N1, H3N2, H3N8, and H7N2)  Canine respiratory coronavirus  *Mycoplasma cynos*  *Streptococcus equi* subspecies *zooepidemicus* | Haemagglutinin fusion protein gene  Hexon gene  Phosphoprotein gene  DNA polymerase gene  Nucleocapsid (SH) gene (GU247050)  Hemagglutinin-neuraminidase gene  N2 gene (JN247597)  Matrix protein 2 (CY049670)  Hemagglutinin-esterase gene  ITS-1 (AF412978)  AroA (FM204884) and 3-phosphoshikimate 1-carboxyvinyltransferase (CP001129) |

Supplemental Table 1: Bacteria and virus gene targets included in the canine respiratory disease panels.
