# Supplementary material for: Frequency of respiratory pathogens and SARS‐CoV‐2 in canine and feline samples submitted for respiratory testing in early 2020
Source: J Small Anim Pract. 2021 Jan 31;62(5):336–42. doi: 10.1111/jsap.13300 (PMC8014115; doi:10.1111/jsap.13300)
Supplement: Supplementary file 2 — Table S2. Bacteria and virus gene targets included in the feline upper respiratory panel. [file JSAP-62-336-s004.docx]

| **Pathogens tested for in the feline upper respiratory disease panel** | **Gene Target** |
| --- | --- |
| *Bordetella bronchiseptica*  *Chlamydophila felis*  Feline calicivirus  Feline herpesvirus type 1  H7N2 avian influenza virus  Influenza A virus (H1N1, H3N2, H3N8, and H7N2)  *Mycoplasma felis* | Haemagglutinin fusion protein gene (AF140678)  Outer membrane protein (AP006861)  ORF 1 (AF109465)  Glycoprotein B  N2 gene (JN247597) and H7 gene (B5865576)  Matrix protein 2 (CY049670)  16S-23S ribosomal RNA intergenic spacer |

Supplemental Table 2: Bacteria and virus gene targets included in the feline upper respiratory panel.
