# Supplementary material for: Frequency of respiratory pathogens and SARS‐CoV‐2 in canine and feline samples submitted for respiratory testing in early 2020
Source: J Small Anim Pract. 2021 Jan 31;62(5):336–42. doi: 10.1111/jsap.13300 (PMC8014115; doi:10.1111/jsap.13300)
Supplement: Supplementary file 3 — Table S3. Counts of individual respiratory microorganisms identified by PCR for feline patients outside of the USA included in the SARS‐CoV‐2 surveillance study in early 2020. [file JSAP-62-336-s005.docx]

Supplemental Table 3: Counts of individual respiratory microorganisms identified by PCR for feline patients outside of the US included in the SARS-CoV-2 surveillance study in early 2020.

| **Region** | Cf | FCV | FHV-1 | Bb | Mf | FInV | InVA |
| --- | --- | --- | --- | --- | --- | --- | --- |
| **Asia** | 6/174 | 57/174 | 34/174 | 4/174 | 39/174 | 0/174 | 0/174 |
| **Europe** | 22/609 | 110/608 | 15/568 | 22/610 | 359/608 | -- | -- |
| **Canada** | 0/5 | 0/5 | 0/5 | 2/5 | 2/5 | -- | 0/5 |

Cf = *Chlamydophila felis;* FCV = feline calicivirus; FHV-1 = feline herpesvirus type 1; Bb = *Bordetella bronchiseptica*; Mf = *Mycoplasma felis*; FInV = H7N2 Influenza; InVA = influenza A
