# Supplementary material for: Frequency of respiratory pathogens and SARS‐CoV‐2 in canine and feline samples submitted for respiratory testing in early 2020
Source: J Small Anim Pract. 2021 Jan 31;62(5):336–42. doi: 10.1111/jsap.13300 (PMC8014115; doi:10.1111/jsap.13300)
Supplement: Supplementary file 4 — Table S4. Counts of individual respiratory microorganisms identified by PCR for canine patients outside of the USA included in the SARS‐CoV‐2 surveillance study in early 2020. [file JSAP-62-336-s001.docx]

| **Region** | Bb | CAV-2 | CHV-1 | CPIV | CPnV | CrCoV | CDV | CInV | InVA | Mc | Se |
| --- | --- | --- | --- | --- | --- | --- | --- | --- | --- | --- | --- |
| **Asia** | 3/35 | 2/35 | 0/35 | 0/35 | 3/35 | 2/35 | 3/35 | 0/35 | 0/35 | 7/35 | 0/35 |
| **Europe** | 5/56 | 0/55 | 0/55 | 1/55 | -- | 0/53 | 0/52 | 0/55 | -- | 11/55 | -- |
| **Canada** | 0/4 | 0/4 | 0/4 | 0/4 | 4/4 | 1/4 | 0/4 | 0/4 | 0/4 | 0/4 | 0/4 |

Supplemental Table 4. Counts of individual respiratory microorganisms identified by PCR for canine patients outside of the US included in the SARS-CoV-2 surveillance study in early 2020.

Bb = *Bordetella bronchiseptica;* CAV-2 = canine adenovirus type 2; CHV-1 = canine herpesvirus type 1; CPIV = canine parainfluenza virus; CPnV = canine pneumovirus; CrCoV = canine respiratory coronavirus; CDV = canine distemper virus; CInV = H3N2 canine influenza virus; InVA = Influenza virus A; Mc = *Mycoplasma cynos*; Se = *Streptococcus equi.*
