# Supplementary material for: Frequency of respiratory pathogens and SARS‐CoV‐2 in canine and feline samples submitted for respiratory testing in early 2020
Source: J Small Anim Pract. 2021 Jan 31;62(5):336–42. doi: 10.1111/jsap.13300 (PMC8014115; doi:10.1111/jsap.13300)
Supplement: Supplementary file 5 — Table S5. Geographic origin of samples included in the year‐over‐year comparison. [file JSAP-62-336-s003.docx]

| **Geographic Region** | **Canine** | | **Feline** | |
| --- | --- | --- | --- | --- |
|  | 2019 | 2020 | 2019 | 2020 |
| **Asia**  South Korea  Singapore | 75  2 | 55  0 | 253  9 | 206  12 |
| **Europe**  Germany  Austria  Italy  Denmark  Norway  Sweden  Finland  Netherlands | 79  7  0  3  7  0  0  0 | 137  4  3  9  9  0  0  3 | 1005  43  44  39  47  17  30  42 | 1142  49  42  56  71  49  41  68 |
| **North America**  United States  Canada | 4779  48 | 4709  71 | 3393  80 | 3129  136 |
| **Total** | 5000 | 5000 | 5000 | 5000 |

Supplemental Table 5: Geographic origin of samples included in the year-over-year comparison.
